# Post-mortem brain analyses of the Lothian Birth Cohort 1936: extending lifetime cognitive and brain phenotyping to the level of the synapse

**DOI:** 10.1186/s40478-015-0232-0

**Published:** 2015-09-04

**Authors:** Christopher M. Henstridge, Rosemary J. Jackson, JeeSoo M. Kim, Abigail G. Herrmann, Ann K. Wright, Sarah E. Harris, Mark E. Bastin, John M. Starr, Joanna Wardlaw, Thomas H. Gillingwater, Colin Smith, Chris-Anne McKenzie, Simon R. Cox, Ian J. Deary, Tara L. Spires-Jones

**Affiliations:** Centre for Cognitive and Neural Systems, University of Edinburgh, 1 George Square, Edinburgh, EH8 9JZ UK; Centre for Integrative Physiology, University of Edinburgh, Hugh Robson Building, George Square, Edinburgh, EH8 9XD UK; Centre for Cognitive Ageing and Cognitive Epidemiology, University of Edinburgh, 7 George Square, Edinburgh, EH8 9JZ UK; Medical Genetics Section, University of Edinburgh Centre for Genomic and Experimental Medicine and MRC Institute of Genetics and Molecular Medicine, Western General Hospital, Edinburgh, EH4 2XU UK; Centre for Clinical Brain Sciences, University of Edinburgh, Chancellor’s Building, 49 Little France Crescent, Edinburgh, EH16 4SB UK; Geriatric Medicine Unit, University of Edinburgh, Western General Hospital, Edinburgh, EH4 2XU UK; Department of Psychology, CCACE, University of Edinburgh, 7 George Square, Edinburgh, EH8 9JZ UK; Euan MacDonald Centre for Motor Neurone Disease Research, University of Edinburgh, Chancellor’s Building, 49 Little France Crescent, Edinburgh, EH16 4SB UK

## Abstract

**Introduction:**

Non-pathological, age-related cognitive decline varies markedly between individuals andplaces significant financial and emotional strain on people, their families and society as a whole.Understanding the differential age-related decline in brain function is critical not only for the development oftherapeutics to prolong cognitive health into old age, but also to gain insight into pathological ageing suchas Alzheimer’s disease. The Lothian Birth Cohort of 1936 (LBC1936) comprises a rare group of people forwhom there are childhood cognitive test scores and longitudinal cognitive data during older age, detailedstructural brain MRI, genome-wide genotyping, and a multitude of other biological, psycho-social, andepidemiological data. Synaptic integrity is a strong indicator of cognitive health in the human brain;however, until recently, it was prohibitively difficult to perform detailed analyses of synaptic and axonalstructure in human tissue sections. We have adapted a novel method of tissue preparation at autopsy toallow the study of human synapses from the LBC1936 cohort in unprecedented morphological andmolecular detail, using the high-resolution imaging techniques of array tomography and electronmicroscopy. This allows us to analyze the brain at sub-micron resolution to assess density, proteincomposition and health of synapses. Here we present data from the first donated LBC1936 brain andcompare our findings to Alzheimer’s diseased tissue to highlight the differences between healthy andpathological brain ageing.

**Results:**

Our data indicates that compared to an Alzheimer’s disease patient, the cognitively normalLBC1936 participant had a remarkable degree of preservation of synaptic structures. However,morphological and molecular markers of degeneration in areas of the brain associated with cognition(prefrontal cortex, anterior cingulate cortex, and superior temporal gyrus) were observed.

**Conclusions:**

Our novel post-mortem protocol facilitates high-resolution neuropathological analysis of the well-characterized LBC1936 cohort, extending phenotyping beyond cognition and in vivo imaging to nowinclude neuropathological changes, at the level of single synapses. This approach offers an unprecedentedopportunity to study synaptic and axonal integrity during ageing and how it contributes to differences in agerelatedcognitive change.

**Electronic supplementary material:**

The online version of this article (doi:10.1186/s40478-015-0232-0) contains supplementary material, which is available to authorized users.

## Introduction

The global number of older, dependent people is projected to reach 277 million by 2050 [1]. According to government figures, more than one-in-four UK inhabitants will be aged 65 or over by the same year [2]. Cognitive decline is a feature of ageing, yet its severity varies dramatically between individuals and a deeper understanding of the underlying factors influencing brain ageing, may render this process open to modification. An important question to address is whether the brain changes observed in healthy older individuals are distinct from changes observed in neuropathologies such as Alzheimer’s disease (AD). For example, synapse loss, amyloid plaques and neurofibrillary tangles are the hallmarks of AD, yet these features can also be observed in the brains of cognitively-healthy older people [3]. However, before we can assess the influence of pathways or proteins in pathological cognitive decline we need a greater understanding of the normal ageing process [4].

Normal cognitive ageing refers to age-related cognitive change with an absence of disease and is thought to be due to a number of underlying factors [4]. As people age, most tissues and bodily functions begin to decline and neurological function is not exempt from this effect. In fact, some age-dependent gene expression changes are evolutionary conserved throughout the animal kingdom from humans to nematode worms, including genes involved in the stress response, mitochondrial function and the immune response [3]. In the brain, changes in neuron number and neurite complexity occur, glial cells become more active, neurotransmitter levels are altered, pigments and proteins begin to accumulate in cells and neurovascular changes become increasingly prevalent [5]. With the introduction of functional MR imaging, a systems view of neuronal activity could be observed for the first time, during the course of brain ageing. This revealed a loss of regional co-ordination in aged brains during higher order cognitive tasks [6]. These effects may be due in part, to altered axonal and/or myelin physiology, as increased incidence of white matter hyperintensities appear to correlate with more severe cognitive decline [7]. However, synapses are now increasingly thought to be essential in the state of cognitive ageing [8]. During normal ageing the brain transcriptome alters and many age-related changes occur in genes involved in synaptic function [9, 10]. Indeed, data from ageing cohorts have revealed that higher levels of presynaptic markers correlate with better cognitive function [11]. Furthermore, a recent UK-based cohort study revealed that genetic variability within synaptic genes, contributes to the variability in general intelligence [12]. These findings add to a growing body of literature suggesting synapses are not only critical for healthy cognitive-ageing, but represent the first neuronal feature to be affected in AD [13, 14].

Large cohort studies that trace cognition through to brain changes at death are a powerful tool for determining the neurobiological contributors to cognitive ageing. Several such studies including the Religious Orders Study and the Rush Memory and Ageing Project have assessed cognitive change in old age and characterized neural tissue post-mortem, providing a wealth of information on potential factors and correlates that may influence elderly cognitive change [15]. However, most ageing studies lack the longitudinal data on individual participant’s cognitive performance from childhood to adulthood which is important for distinguishing which changes correlate with cognitive variation in age are truly age-related as opposed to being effects of other factors such as genetics [16].

The Lothian Birth Cohort of 1936 (LBC1936) is a sample of 1091 people for whom we have childhood cognitive test scores, longitudinal cognitive data during ageing, detailed structural MRI, genome-wide genotyping, and a multitude of other biological and epidemiological data [17]. Furthermore, 173 participants of the cohort have agreed to donate their brains to the study for detailed post-mortem analysis. The cohort’s data have been used to examine determinants of cognitive change between childhood and age 70, and within older age. The LBC1936 research team refers to the age 11 to age 70 cognitive ageing as ‘lifetime cognitive ageing’, and their candidate determinants include genetic, lifestyle, psychosocial and biomedical (including brain imaging factors). The following factors make small contributions to cognitive efficiency in older age after adjusting for childhood cognitive function (which effectively means they contribute positively to lifetime cognitive ageing): being bilingual [18], attaining higher education, [19], having had a more complex occupation [20], having more social support and being less lonely [21], being physically more active [22], having a lower genetic risk of schizophrenia [23] and lacking the e4 allele of the gene for APOE [24]. The LBC1936 study has found several instances in which cross-sectional associations between putative determinants of cognitive ageing and cognitive test scores at age 70 are almost wholly confounded by childhood intelligence test scores. Thus, engaging more in socio-intellectual activities [22], drinking more red wine at moderate levels [25], having a lower body mass index [26], eating a Mediterranean diet [27], having lower blood levels of C-reactive protein [28] and not being infected by the cytomegalovirus [29] are all associated with better cognitive functions at age 70, but these effects are almost nullified after adjusting for intelligence test scores at age 11. With regard to structural brain variables, less relative decline in cognitive function between age 11 and the 70s is associated with better white matter structure [30], having fewer white matter hyperintensities [7], having less brain atrophy [31] and having fewer iron deposits [32]. Even with brain imaging-cognition associations there is some confounding; for example, the cross-sectional association between cognitive function in older age and brain cortical thickness was almost wholly accounted for by cognitive ability test scores at age 11 years [33].

Given the wealth of data available in the LBC1936 study, our aim is to extend post-mortem characterization of each donated brain to the level of the synapse, to document synaptic alterations as a potential substrate for the neuropathological underpinnings of cognitive change during ageing. Until recently it was not possible to accurately quantify synaptic and axonal structure and protein composition in human post-mortem brain sections. However, here we describe a modified tissue processing procedure at autopsy, which allows the preservation of human brain ultrastructure for super-resolution electron microscopy and array tomography. We have pioneered the use of this technique for human tissue and published a detailed protocol to allow others to utilize this approach [34], however until now this technique has never been used to study non-pathological cognitive ageing. This approach will allow detailed analysis of human brain at the level of individual synapses and assessment of numerous proteins (pathological or physiological) at these synapses. Ultimately, this will allow us for the first time to characterize, within an individual, youth intelligence to older age-related cognitive change and the underlying macroscopic and microscopic alterations within the cells and synapses of the brain at post-mortem.

In summary, here we present methods and pilot data from the first donated LBC1936 brain tissue and describe in detail the multi-disciplinary post-mortem approach to be used for the remaining cohort, which we expect will ultimately generate a wealth of information on brain health and underlying pathology.

## Materials and methods

### Cognitive testing

When recruited into the study at age 70 years, all LBC1936 participants underwent a range of cognitive tests covering reasoning, memory, executive function and speed of information processing, as described previously [35]. Included in the range of tests, was the same Moray House Test No.12 they took at the age of 11. This is a well-validated test of general intelligence.

### MRI acquisition and processing

All MRI data were acquired using a GE Signa Horizon HDxt 1.5 T clinical scanner (General Electric, Milwaukee, WI) equipped with a self-shielding gradient set (33 mT/m maximum gradient strength) and manufacturer supplied eight-channel phased-array head coil. As described in detail in [36] the examination comprised the following whole-brain structural sequences acquired with contiguous slice locations: T2- (T2W), T2*- (T2*W) and FLAIR-weighted axial scans (80, 80 and 40 slices respectively) and a high-resolution T1-weighted (T1W) volume sequence acquired in the coronal plane (160 slices). The field-of-view was 256 × 256 mm in all cases, with voxel dimensions of 1 × 1 × 2 mm for T2W and T2*W, 1 × 1 × 4 mm for FLAIR, and 1 × 1 × 1.3 mm for T1W. These data were then converted from DICOM (http://dicom.nema.org) to NIfTI-1 (http://nifti.nimh.nih.gov/nifti-1) format and registered together using FSL tools (http://www.fmrib.ox.ac.uk/fsl) to allow visualization.

### Post-mortem analysis

Use of human tissue for post-mortem studies has been reviewed and approved by the Edinburgh Brain Bank ethics committee and the ACCORD medical research ethics committee, AMREC (ACCORD is the Academic and Clinical Central Office for Research and Development, a joint office of the University of Edinburgh and NHS Lothian). The Edinburgh Brain Bank is a Medical Research Council funded facility with research ethics committee (REC) approval (11/ES/0022). Tissue from three donors was used for this study and their details are found in Table 1.

**Table 1:** Post-mortem details of the tissue donors in this study

	LBC	AD	MND
Sex	Female	Male	Male
Age	77	57	50
PM Delay	75 h	58 h	89 h
Brain Weight (g)	1320	1200	1350
Brain pH	6.5	5.9	6.5
Clinical Notes	Small Vessel Disease	Alzheimer’s Disease	Motor Neuron Disease
	Braak I	Braak VI	
	Old Microinfarcts		
	Cerebral Amyloid Angiopathy (CAA)		

**Fig. 1:**
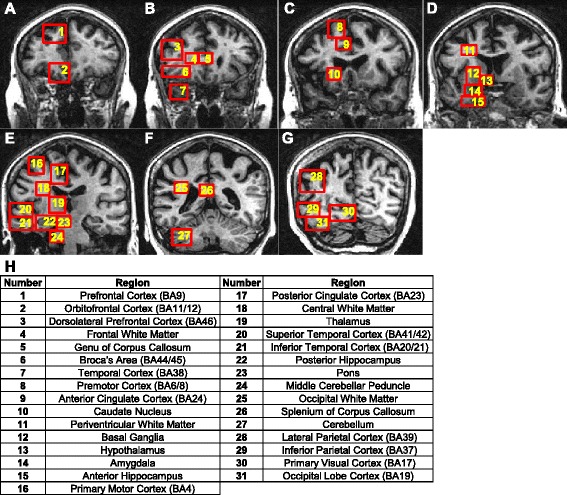
MRI showing the location of brain regions sampled for post-mortem analysis. FLAIR-weighted, coronal MR images from anterior to posterior (**a**-**g**) of the LBC1936 participant’s brain. Post-mortem, regions were collected for analysis as highlighted by the numbered boxes. **h** Numbered boxes correspond to the brain regions labelled in the table, with corresponding Brodmann Area for each cortical region provided

At post-mortem the brain was removed as detailed previously [37] and cut into coronal slices while still unfixed. Regions of interest were then dissected from each coronal slice. As can be seen in Fig. 1, 31 regions are marked for dissection however some of these are subdivided into other regions of interest within that block. The hippocampus is divided into Entorhinal Cortex, CA1 and Dentate Gyrus, the Basal Ganglia is separated into the Substantia Nigra, Putamen and Internal Capsule and BA6/8, BA23 and BA39 also contain underlying subcortical white matter blocks. This adds another 7 regions, bringing the total to 38. Each region of interest from one hemisphere is then processed for paraffin embedding or frozen for biochemistry. Samples from the other hemisphere are placed in small bijous containing 0.1 M Phosphate Buffer (PB) and dissected into smaller segments. These samples are processed for array tomography [34] or electron microscopy and the remaining tissue is snap frozen on dry ice for biochemistry.

### Biochemistry

Chemicals were obtained from Sigma-Aldrich UK unless otherwise noted. Synaptoneurosomes and crude homogenates were prepared according to [38]. Briefly, 200 mg of fresh-frozen human brain tissue was homogenized in a glass dounce homogenizer with 1 mL ice-cold buffer A (25 mmol/L HEPES pH 7.5, 120 mmol/L NaCl, 5 mmol/L KCl, 1 mmol/L MgCl_2_, and 2 mmol/L CaCl_2_), supplemented with 2 mmol/L dithiothreitol (DTT), protease inhibitors (Roche complete mini), and phosphatase inhibitors (Calbiochem #524629). The homogenate was passed through 2 layers of 80-μm nylon filters (Millipore, Watford, UK), and a 200-μL aliquot of the filtered homogenate was saved. The saved aliquot was mixed with 200 μL water and 60 μL 10 % SDS, to prepare the crude homogenate.

To prepare synaptoneurosomes, the remainder of the homogenate was passed through a 5-μm Durapor membrane filter (Millipore) to remove large organelles and nuclei and centrifuged at 1000 g for 5 mins. The non-synaptic supernatant containing cytoplasmic proteins was removed, and the pellet was washed once with buffer A and centrifuged again, yielding the synaptoneurosome pellet. This was suspended in 400 μL of Buffer B (50 mmol/L Tris [pH 7.5], 1.5 % SDS, and 2 mmol/L DTT) and boiled for 5 mins. 10 % SDS was added to the supernatant fraction to bring the concentration to 1.5 % SDS and this was boiled for 5 min.

Protein levels were analyzed in each sample using a BCA protein assay (Thermo Fisher Scientific, Loughborough, UK). 5 μg of protein was loaded into precast NuPAGE 4–12 % Bis-Tris polyacrylamide 15 well gels (Invitrogen, Paisley, UK) alongside a molecular weight marker (Li-Cor, Cambridge, UK). Electrophoresis was performed at 100 V for 2 h. Proteins were electro-transferred to nitrocellulose membrane (Bio-Rad, Hemel Hempstead, UK) at 30 V for 1.5 h using the XCell II™ Blot Module system (Invitrogen, Paisley, UK) in tris-glycine transfer buffer. Membranes were incubated in 10 ml Odyssey blocking buffer (Li-Cor, Cambridge, UK) diluted 1:1 with Phosphate Buffered Saline (PBS) for 1 h and then incubated with primary antibodies overnight. Primary antibodies used for western blots are shown in Table 2. After washing six times for 5 min in PBS/0.1 % Tween-20 solution, the membranes were incubated for 1 h with the appropriate 680 and 800 IR dye secondary antibodies (1:50000, LI-COR Biosciences). The membranes were washed again in PBS/0.1 % Tween-20 solution. The membranes were imaged using an Odyssey infrared imaging system, corrected for background, and analyzed using Odyssey software (LI-COR Biosciences). For figure preparation the imaged blots were cut to only show the bands that were used for the densitometry analysis. Molecular weights are provided in the figure legends.

**Table 2:** Primary antibody information

Western blots				
Antibody	Company	Code	Dilution	
PHF1	Peter Davies		1:500	
Tau13	Covance	MMS-520R-500	1:2000	
GAPDH	Abcam,	Ab8245	1:2000	
Beta-actin	Abcam	Ab8226	1:2000	
Synaptophysin	Abcam	Ab8049	1:5000	
Beta-III-tubulin	Abcam	Ab18207	1:1000	
MBP	AbD Serotec	MCA409s	1:500	
Histone	Abcam	Ab1791	1:1000	
VDAC1/Porin	Abcam	Ab34726	1:500	
GluN2B	BD Biosciences	610416	1:500	
Synapsin	Millipore	AB1543P	1:20000	
Neuropathology				
Antibody	Company	Code	Dilution	Pre-treatment
Beta Amyloid (BA4)	Dako	M087201-2	1:100	98 % formic acid 5 min
Alpha Synuclein	Life Technologies	32-8100	1:200	Pressure cooker/formic acid
TDP-43	2B Scientific	CAC-TIP-PTD-MO1	1:4000	Pressure cooker/citric acid
pTau (AT8)	Thermo	MN1020	1:2500	None
Ubiquitin	Dako	Z0458	1:500	Pressure cooker/citric acid
GFAP	Dako	Z0334	1:800	None
CD68	Dako	M0876	1:100	Pressure cooker/citric acid
Array tomography				
Antibody	Company	Code	Dilution	Secondary antibody
AW7	Dominic Walsh		1:1000	Donkey α Rabbit – AF488
Synaptophysin	Abcam	Ab8049	1:50	Donkey α Mouse – AF594
ApoE	Abcam	Ab7620	1:50	Donkey α Goat – AF647
PSD95	Abcam	Ac12093	1:50	Donkey α Goat – AF488

### Neuropathology

Fresh post-mortem tissue blocks (approximately 1 cm^3^) from our regions of interest were fixed in 10 % formalin for a minimum of 24 h. Tissue was then dehydrated in an ascending series of alcohol (70–100 %), followed by three xylene washes, all for 4 h each. Next, three paraffin waxing stages (5 h each) were performed to ensure full penetration of the embedding wax and finally these blocks were allowed to cool. Tissue sections were cut on a Leica microtome at 13 μm and collected on glass slides. All sections were dried at 40 °C for at least 24 h before staining.

Immunohistochemistry was performed using standard protocols, enhanced using the Novolink Polymer detection system and visualized using 3,3′-Diaminobenzidine (DAB) as chromogen. See Table 2 for antibody information. Slides were finally counterstained with hematoxylin for 30 s to stain cell nuclei. Images were scored semi-quantitatively according to the estimated level of pathology. Additional file 1: Figure S1 contains example images for each stain and pathology score.

### Burden quantification

All sections stained for β-amyloid and GFAP were assessed for plaque and astrocytic burden respectively using Stereo Investigator. Cortical grey matter was outlined in each section and immune-positive objects identified using an automated colour-based thresholding algorithm. The area of immuno-positive cortex was expressed as a percentage of total cortex in each brain region.

### Cortical thickness

Hematoxylin and eosin stained cortical sections were used to measure cortical thickness. This stain provides a clear demarcation between grey and white matter. Grey matter thickness was calculated at eight randomly selected points throughout each section. Thickness was measured from the pial surface to the border of grey/white matter, below cortical layer 6. All eight measurements were averaged to give a cortical thickness for each brain region.

### Neuron and microglia density

Neuron and microglia densities were generated within the grey matter of each cortical section, stained with CD68. Using a stereological optical dissector approach (described in [39], approximately 250 fields of view were randomly scattered across the cortical grey matter. Within each field of view a dissector grid of 200 μm × 200 μm was applied and all CD68-positive cells within the grid and touching the acceptable boundaries were counted. Neurons were identified based on the presence of large oblong nuclei and were counted within each dissector grid. Total cell counts within all grids were divided by total imaged volume to generate densities of cells per mm^3^. Finally, CD68-positive cell soma cross-sectional area was measured to ensure changes in cell size were not influencing our total counts. We discovered no change in CD68-positive cell size between cases when more than 300 cells per case from two cortical regions were analysed (data not shown).

### Electron microscopy

Brain samples were prepared for electron microscopy as previously outlined in detail [34]. Briefly, fresh post-mortem samples, stored in 0.1 M PB were trimmed into small cortical blocks and fixed in 4 % paraformaldehyde and 2.5 % glutaraldehyde in 0.1 M PB for 48 h. Blocks were washed twice in 0.1 M PB, then cut at 70 μm with a vibratome. Sections were treated with osmium tetroxide (1 % in 0.1 M PB) for 30mins (protected from light) and dehydrated in an ascending series of ethanol and propylene oxide, before embedding in Durcupan resin. During dehydration the sections were treated with uranyl acetate (1 % in 70 % ethanol) for 40mins in the dark. Durcupan resin was polymerized for 48 h at 56 °C. Small regions of interest were cut from the Durcupan-embedded sections and glued onto Durcupan blocks, before cutting 70 nm ribbons with an ultracut microtome (Leica) using a Jumbo Histo Diamond Knife (Diatome, Hatfield, PA). Ribbons were collected on grids and stained with lead citrate before imaging on a JEOL JEM-1011 transmission electron microscope (TEM) with Hamamatsu ORCA digital camera. For synapse analysis, an average of 16 images per region (range 10–28) were taken at 20,000× magnification in a systematic, random fashion from blocks from BA17, BA24, BA41/42, BA44/45, BA46, BA6/8, BA9, entorhinal cortex, and CA1 stratum radiatum from the LBC1936 and AD cases.

For white matter analysis, an average of 5 images at 8,000× magnification were taken in a systematic random fashion from 4 white matter regions of the LBC1936 case (BA39 subcortical white matter, BA6/8 subcortical white matter, genu of the corpus callosum, and periventricular white matter). G-ratios were measured as described previously [40]. TEM images were coded for blind analysis. In grey matter images, synapses were defined by the presence of at least 3 synaptic vesicles in the presynaptic terminal and a clear postsynaptic density. For each synapse, the following measurements were made: apposition length, number of presynaptic vesicles, pre and postsynaptic terminal cross-sectional areas. Each synapse was also classified as excitatory or inhibitory (based on intensity of PSD, synaptic vesicle morphology, and cleft width) and the presence of multivesicular bodies (MVB), mitochondria, spine apparatus (in the postsynapse), perforated postsynaptic density (PSD), and dark degenerating terminals was noted. In white matter images, diameters of axons and axon + myelin sheaths were measured and the presence of mitochondria was noted. A total of 1600 axons were measured.

### Array tomography

Brain samples were prepared for array tomography as previously outlined in detail [34]. A summary flowchart of the steps involved is provided in Additional file 2: Figure S2. Briefly, fresh post-mortem samples were trimmed into small cortical blocks and fixed in 4 % paraformaldehyde for 3 h. Samples were dehydrated through ascending ethanol washes and embedded in LR White resin. Tissue blocks were cut into ribbons of 70 nm sections and collected on coverslips. Ribbons were immunostained on “day1” with antibodies against Synaptophysin, AW7 and ApoE overnight (see Table 2 for antibody details). Sections were then developed with fluorescently-labelled secondary antibodies and images obtained along the ribbon using a Zeiss axio Imager Z2 epifluorescent microscope equipped with a CoolSnap digital camera and AxioImager software with array tomography macros (Carl Zeiss, Ltd, Cambridge UK). High-resolution images were obtained with a 63 × 1.4 NA Plan Apochromat objective. Coverslips were washed twice in TBS and stripped of all antibodies with a 10 min wash in stripping buffer (0.2 M NaOH and 0.02 % SDS in TBS). The staining protocol was then repeated (“day2”) with antibodies against PSD-95 (see Table 2 for antibody details). Negative controls lacking primary antibody were run alongside to ensure the specificity of the protocol. These were always blank (data not shown). Images from “day1” and “day2” were combined, aligned, and regions of interest (crops) chosen in the neuropil. These crops were coded for blind analysis and thresholded with automated algorithms in ImageJ. Synaptic puncta counts and volumes were generated using our own MATLAB scripts and analysis of co-localization with pathological proteins expressed as a percentage of total synapses. All ImageJ macros and MATLAB scripts will be made available upon request.

### Statistics

GraphPad Prism was used to test array tomography data for normality (Kolmogorov-Smirnov test), following which parametric (*t*-test or ANOVA) or non-parametric (Kruskal-Wallis or Mann–Whitney) tests were performed as required. SPSS was used to compute statistics for electron microscopy data. Normality of data was tested with a Shapiro-Wilkes test. Normally distributed data were analyzed with parametric statistics and non-normal data with non-parametric tests as required. Significance was reported when *P* < 0.05.

## Results

### Cognitive summary of the first LBC1936 brain donor

The LBC1936 participant scored either 28 or 29 out of 30 on the Mini-Mental State Examination on three occasions of testing at about 70, 73, and 76 years, indicative of no substantial cognitive decline. The participant’s scores on the Moray House Test (verbal reasoning; age 11, 70 and 76), Wechsler Logical Memory (verbal declarative memory; age 70, 73, and 76), and Wechsler Digit Symbol (processing speed; age 70, 73 and 76) were between 0.5 and 1.0 standard deviations (SD) higher than their LBC1936 contemporaries at age 11 or 70, but only between 0 and 0.5 SD higher at age 76. On Wechsler Matrix Reasoning (non-verbal reasoning; ages 70, 73, and 76) the participant’s score was consistently about 0.25 SD below the LBC1936 cohort mean. In summary, the participant exhibited no cognitive decline and generally scored above the average LBC1936 score in most tests, with some decline in that advantage at the last round of tests. At the third test, the participant mentioned suffering a small stroke since the second wave (aged 73) and that following the stroke she had noticed minor memory problems. The participant died 8 months after the final cognitive test.

### Structural MRI

**Fig. 2:**
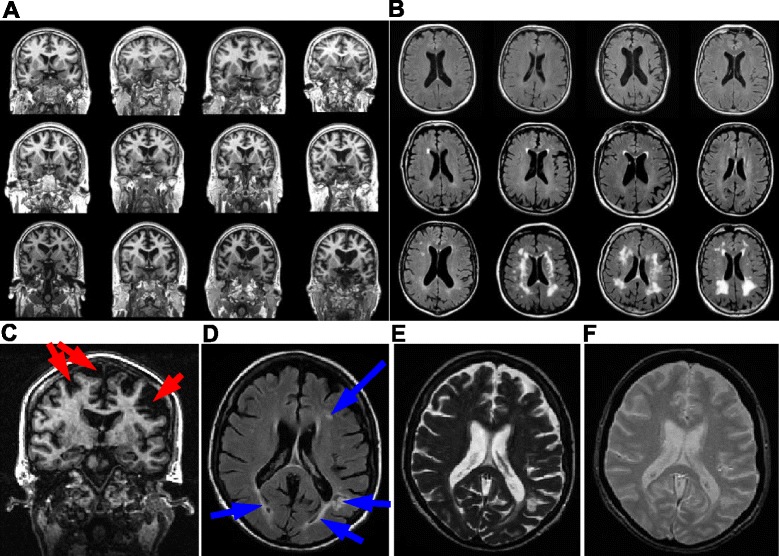
*In vivo* MRI of the LBC1936 cohort reveals a broad spectrum of pathology. **a** FLAIR-weighted, coronal MR images from 12 individual LBC1936 participants, highlighting the range of gross brain pathology observed, such as ventricular enlargement and cortical atrophy. Pathology worsens from the top left to bottom right. **b** T2-weighted, horizontal MR images depicting the range of white matter hyperintensities observed in the LBC1936. Most are periventricular, but some appear to be subcortical. Again, pathology worsens from the top left to bottom right. **c** Coronal T1-weighted image from the LBC1936 participant who donated their brain for post-mortem study, revealing some cortical atrophy (red arrows). **d** Horizontal axial FLAIR-weighted MR image, revealing white matter hyperintensities (blue arrows). **e** Horizontal T2-weighted and GRE-weighted (**f**) MR images reveal generally good brain structure

To illustrate how the degree of cortical thinning and ventricular enlargement present in our subject compared with that seen across the entire LBC1936, Fig. 2a shows representative coronal images from twelve participants, arranged by degree of cortical thinning and ventricular enlargement from top left to bottom right. Cortical thinning is a common feature of brain ageing [41] and in Fig. 2c it can be seen that the LBC1936 case here also exhibits some localized cortical thinning, highlighted by red arrows. The level of cortical thinning was described as being within normal limits for age, by the study’s radiologist. White matter hyperintensities are a common feature of brain ageing and are more common in patients with cardiovascular problems and neurodegenerative diseases such as Alzheimer’s [42]. They tend to be found in the deep white matter tracts of the brain [42] and as can be seen in Fig. 2b, their presence varies strikingly between the age-matched, non-demented LBC1936 cohort participants. The LBC1936 participant in this study exhibited a number of mild periventricular and subcortical white matter hyperintensities as highlighted by the blue arrows in Fig. 2d. No microhaemorrhage or mineralization was noted in the T2-weighted (Fig. 2e) or GRE-weighted scans (Fig. 2f). In fact, the only other feature of note from the imaging studies was the remains of small old infarcts in the left frontal and lateral occipital lobes.

### Outline of post-mortem studies

**Fig. 3:**
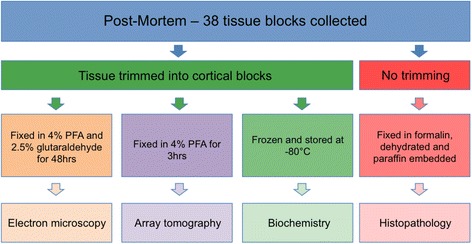
Summary of post-mortem tissue processing. Flowchart showing the numerous processing techniques employed, allowing us to gain cellular and subcellular information on brain integrity

Post-mortem analysis can be performed on up to 38 freshly dissected brain regions (Fig. 3). Once removed, these fresh tissue blocks will be processed in numerous ways to allow comprehensive characterization, from gross biochemistry to super-resolution electron microscopy.

### Neuropathology

**Fig. 4:**
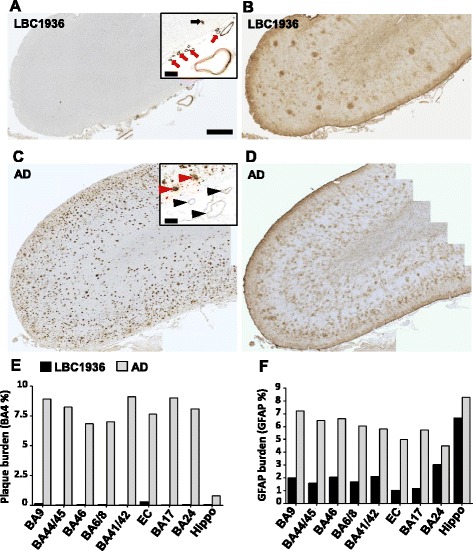
Stereological assessments of amyloid plaque burden and gliosis. **a** Premotor cortex (BA6/8) from the LBC1936 brain contains no amyloid plaques within the cortical tissue. However, some blood vessels within the cortex (insert, black arrow) and around the pial surface (insert, red arrows) exhibit strong amyloid labelling. **b** LBC1936 BA6/8 region exhibits a dense network of GFAP staining in layer 1 of the cortex, with small patches of GFAP-positive cells scattered through the other cortical layers. **c** BA6/8 from the AD case contains a very high amyloid burden throughout the cortex. Despite the presence of cortical plaques (insert, red arrowheads) the pial vessels were largely devoid of labelling (insert, black arrowheads). **d** GFAP-positive cells were found throughout the AD BA6/8 cortical region and often found around plaque-like structures. Large scale bars = 1 mm, insert scale bars = 0.2 mm. **e** Histogram showing amyloid burden in eight cortical regions and the hippocampus in the LBC1936 case (black bars) and the AD case (grey bars). **f** Histogram showing GFAP burdens in eight cortical regions and the hippocampus from the LBC1936 and AD cases

The neuropathological correlates of cognitive decline in healthy ageing (if any) are not well understood. All brain regions were immuno-stained with antibodies against proteins known to be involved in neurodegenerative diseases associated with cognitive decline. Semi-quantitative scoring of the immunostaining revealed a very low level of pathology in the non-demented LBC1936 brain compared to the AD case (Table 3). Braak scoring revealed the LBC1936 had mild early stage tau pathology (Braak stage I), whereas the AD case had much more severe pathology (Braak stage VI). To allow more meaningful association between cognitive status and the extent of pathology in the brain of our LBC1936 subjects, we used stereological approaches to quantify some of the pathological stains. The two major pathological hallmarks of AD are extracellular β-amyloid plaques and intracellular tau-positive neurofibrillary tangles. However it is known that these can also appear in cognitively normal aged brains [43, 44]. The LBC1936 brain contained a very small number of amyloid plaques, mostly restricted to the entorhinal cortex (Fig. 4a, e). Figure 4a shows the premotor cortex (BA6/8) devoid of cortical amyloid plaques, yet quite distinct amyloid staining of some blood vessels within and on the surface of the cortex (Fig. 4a insert), revealing that the participant suffered from cerebral amyloid angiopathy (CAA). This vascular amyloid deposit is found in approximately 50 % of people over the age of 70 years and is not dependent on more global Alzheimer’s-like deposition throughout the grey matter [45]. This was in striking contrast to the AD brain, which contained a high plaque burden throughout the premotor cortex (Fig. 4c) and more globally throughout the brain (Fig. 4e). Interestingly, despite this massive amyloid burden, the blood vessels appeared mostly free of CAA (Fig. 4c insert).

**Table 3:** Semi-quantitative scoring of neuropathological markers

LBC	AD			LBC	AD		
Region	Stain	Score		Region	Stain	Score	
BA9	TDP43	-	++	BA41/42	TDP43	-	++
	pTAU	-	+++		pTAU	-	+++
	BA4	+	+++		BA4	+	+++
	a-Syn	-	-		a-Syn	-	-
	GFAP	+	+++		GFAP	++	+++
	CD68	+	+++		CD68	+	+
	UBIQ	+	+++		UBIQ	+	+++
BA44/45	TDP43	+	+	EC	TDP43	+	++
	pTAU	-	+++		pTAU	+	+++
	BA4	-	+++		BA4	++	+++
	a-Syn	-	-		a-Syn	-	-
	GFAP	+	++		GFAP	+	+++
	CD68	++	++		CD68	+	++
	UBIQ	+	+++		UBIQ	++	+++
BA46	TDP43	+	++	BA17	TDP43	+	++
	pTAU	-	+++		pTAU	-	+++
	BA4	-	+++		BA4	+	+++
	a-Syn	-	-		a-Syn	-	-
	GFAP	+	++		GFAP	+	+++
	CD68	+	++		CD68	+	+
	UBIQ	+	+++		UBIQ	+	+++
BA6/8	TDP43	+	++	BA24	TDP43	+	++
	pTAU	-	+++		pTAU	-	+++
	BA4	+	+++		BA4	-	+++
	a-Syn	-	-		a-Syn	-	-
	GFAP	+	+++		GFAP	+	++
	CD68	+	+++		CD68	+	++
	UBIQ	+	+++		UBIQ	+	+++

BA9 = Prefrontal cortex, BA44/45 = Broca’s area, BA46 = Dorsolateral Prefrontal cortex, BA6/8 = Premotor cortex, BA41/42 = Superior Temporal cortex, EC = Entorhinal cortex, BA17 = Primary Visual cortex, BA24 = Anterior Cingulate cortex. “-” = no pathology, “+” = mild pathology, “++” = moderate pathology, “+++” = strong pathology. Example images for each score are found in Additional file 1: Figure S2

**Fig. 5:**
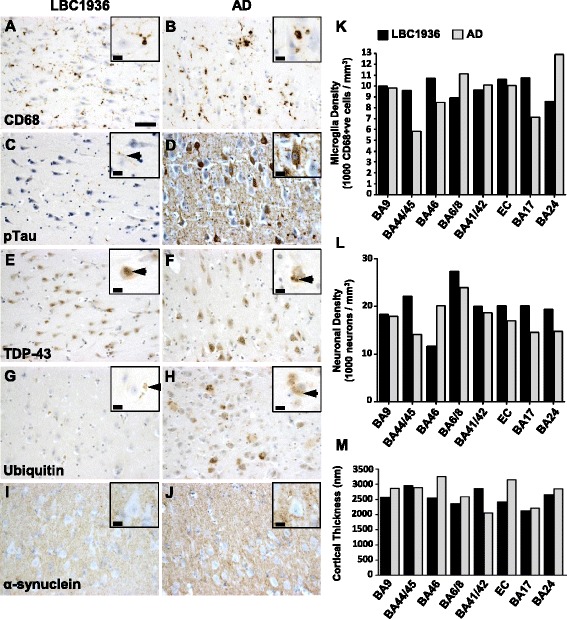
Stereological assessments of brain pathology and integrity. Representative images of microglial (CD68; **a** + **b**), phosphorylated-tau (**c** + **d**), TDP-43 (**e** + **f**), ubiquitin (**g** + **h**) and α-synuclein (**i** + **j**) staining in the hippocampus (CA1) from LBC1936 (**a**, **c**, **e **,**g**, **i**) and AD (**b**, **d**, **f**, **h**, **j**) brains. Large scale bar = 50 μm, insert scale bars = 10 μm. **k** Histogram showing neuronal densities in eight cortical regions from the LBC1936 (black bars) and AD (grey bars) brains. **l** Histogram showing microglial densities (CD68-positive cells) in eight cortical regions from the LBC1936 and AD brains. **m** Histogram of cortical thickness measurements from eight cortical regions in the LBC1936 and AD brains

The other pathological hallmark of Alzheimer’s disease is tau pathology and in the hippocampal CA1 region from the LBC1936 brain, only a few rare tau-positive neurites (Fig. 5c) were found. As expected, the AD hippocampus was full of tau-positive tangles within neuronal somata and dystrophic neurites (Fig. 5d).

Glia are integral for normal brain function and are found throughout the brain under normal conditions. However, gliosis is a common feature of many neuropathologies [46] and as shown in Fig. 4b, d, f the astrocytic burden in the AD brain was higher than the LBC1936 brain in all regions analyzed. Interestingly, microglial densities were much more variable between cases with higher densities found in some cortical regions of the LBC1936 brain (Fig. 5a, b, k). Transactive response DNA binding protein 43 kDa (TDP-43) is a transcriptional repressor protein linked to fronto-temporal dementia (FTD) and Amyotrophic Lateral Sclerosis (ALS) [47]. TDP-43 is normally found in the neuronal nucleus but in patients with FTD it spreads into the cytoplasm in a phosphorylated and ubiquitinated form [47]. The staining of TDP-43 in the LBC1936 hippocampus appeared to be mostly nuclear (Fig. 5e), and this pattern was similar in all other regions (Table 3), however in some cortical regions, translocation of TDP-43 was apparent in a small population of neurons as the strong nuclear labeling was replaced with diffuse somatic labeling. The AD brain exhibited dense cytoplasmic staining and a complete clearing of nuclear staining in a large subset of neurons in many cortical regions (Table 3) and the hippocampal CA1 region (Fig. 5f). Ubiquitin is a small regulatory protein that plays an important role in the post-translational modification of numerous proteins, the addition of which often leads to protein aggregation or degradation [48]. There was a striking difference in the level and pattern of ubiquitin staining between both cases with mostly glial staining in the LBC1936 hippocampus (Fig. 5g) and very strong somatic labelling of many neurons in the AD hippocampus (Fig. 5h). This pattern was found throughout the brain regions analysed (Table 3). Alpha-synuclein is highly expressed in the brain and is an integral protein of the presynaptic terminal. Insoluble fibrils are often found in neuronal somata in diseases such as multiple-system atrophy, Parkinson’s disease, Alzheimer’s disease and dementia with Lewy bodies [49]. However, no distinct alpha-synuclein aggregates were found in either the LBC1936 (Fig. 5i) or the AD hippocampus (Fig. 5j). This pattern was similar throughout the brain of each case (Table 3). Semi-quantitative scoring of all stains in all regions analyzed is summarized in Table 3.

As Alzheimer’s disease progresses, neurons die and brain atrophy occurs. This is highlighted in our neuron density counts, revealing a generally higher neuronal density in most regions of the LBC1936 brain (Fig. 5l). Neuron density values can be influenced by cortical thickness, as the packing of cells can increase in smaller volumes. In most regions, the cortical thickness was similar between cases, however in EC and BA46 the AD cortex appears thicker, but thinner than the LBC1936 cortex in BA41/42 (Fig. 5m).

In summary, the neuropathological assessment of the LBC1936 brain suggests good structural integrity although the brain does show a mild neuropathological burden, which maybe suggestive of early neurodegenerative processes.

### Biochemistry

**Fig. 6:**
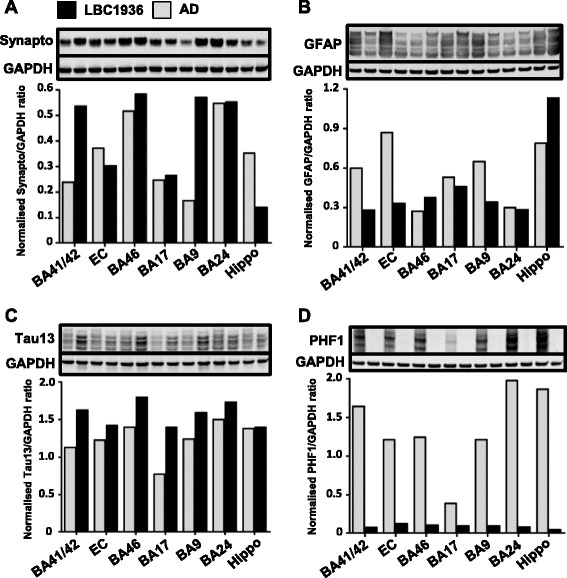
High-throughput assessments of brain integrity and pathology using crude homogenate western blotting. **a** Representative synaptophysin (40 kDa) western blot of seven LBC1936 homogenates (black bars) and seven AD samples (grey bars). **b** GFAP (35-50 kDa) western blot of LBC1936 and AD samples. **c** Representative total tau (Tau13; 45–60 kDa) blot, with LBC1936 and AD samples. **d** Phosphorylated-tau (PHF1; 50–65 kDa) western blot of LBC1936 and AD samples. GAPDH (36 kDa) was run as a loading control for all experiments. Histogram represents the mean of three experimental repeats and the bars represent the quantification of the bands directly above

**Fig. 7:**
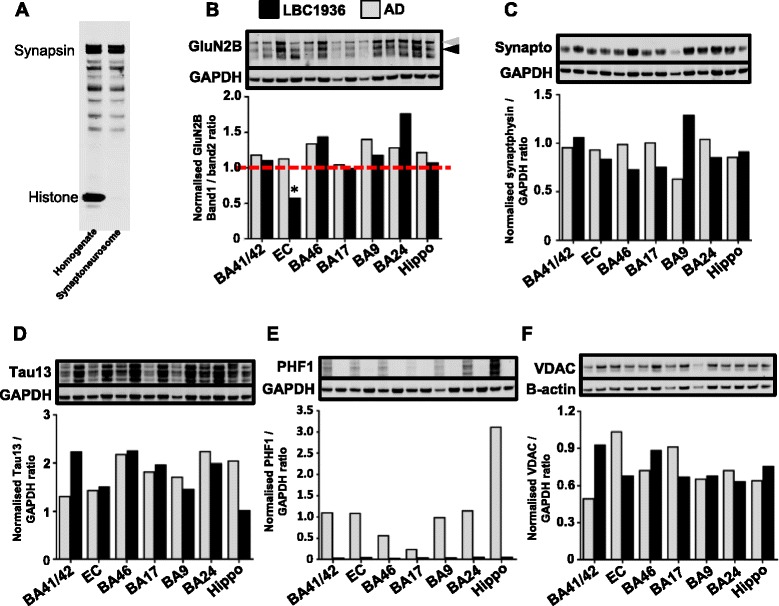
Using synaptoneurosomes to enrich and analyze synaptic proteins by western blotting. **a** Representative enrichment blot showing the exclusion of nuclear histone (17 kDa) from the synaptoneurosome preparation and retention of synapsin (40-80 kDa). **b** GluN2B western blot from the LBC1936 (black bars) and AD (grey bars) preparations. To assess protein integrity and control for post-mortem degradation [52], band2 (black arrow; 150 kDa) was divided by band1 (grey arrow; 170 kDa) to generate a ratio, and a value ≥1 (red dashed line) is achieved by all samples except the LBC1936 EC (asterix). **c** Representative synaptophysin (40 kDa) blot of LBC1936 synaptoneurosomes and AD samples. **d** Representative total tau (Tau13; 45–60 kDa) blot of LBC1936 samples and AD samples. **e** Phosphorylated-tau (PHF1; 50–65 kDa) blot shows almost exclusive expression in the AD samples compared to the LBC1936 synapses. **f** Representative VDAC (29–32 kDa) blot of LBC1936 and AD synaptoneurosomes. GAPDH (36 kDa) or β-actin (42 kDa) was run as a loading control. Histogram represents the mean of three experimental repeats and the bars represent the quantification of the bands directly above

To assess the levels of synaptic proteins, blots of crude homogenate were stained for the pre-synaptic marker synaptophysin. Synaptophysin staining revealed a decrease in synapses in most regions of the AD brain compared to the LBC1936 (Fig. 6a), except the EC and hippocampus (Fig. 6a). GFAP staining revealed a group of bands due to the expression of multiple GFAP isoforms in human brain [50]. In many cortical regions the AD samples expressed higher levels of GFAP than the LBC1936 case (Fig. 6b) as expected from our stereological burden counts (Fig. 4), however in BA46 and the hippocampus the levels were higher in the LBC1936 sample. Tau blots revealed a higher level of this neuronal protein in all regions of the LBC1936 brain compared to AD (Fig. 6c), possibly due to neuronal loss observed in advanced AD [51]. Strikingly, the phosphorylated form of tau was present in all regions of the AD brain, but virtually absent in the LBC1936 samples (Fig. 6d). Collectively, these blots highlight low levels of AD-like pathology in the non-demented LBC1936 brain, however whole cell homogenates can only provide an estimate of synaptic proteins. Many non-synaptic proteins and synaptic proteins that have not yet trafficked to the synapse are present in whole cell homogenates, providing an inaccurate measure of localized synaptic proteins. To provide more accurate synaptic measurements, synaptoneurosome preparations were generated to isolate the synaptic components from the whole homogenate (Fig. 7a). To assess the quality of our preparations we used a recently established method for analysing synaptic protein integrity in brain homogenates [52]. This revealed all our samples except the LBC1936 EC contained good protein integrity (Fig. 7b). These new preparations reveal a more subtle change (if any) in synaptophysin levels in the remaining synapses between the LBC1936 and AD samples (Fig. 7c). Total tau blots revealed the presence of this protein at synapses and showed that again in most regions, similar levels were observed in the LBC1936 samples compared to AD synaptoneurosomes (Fig. 7d). Toxic forms of tau are thought to accumulate in axon terminals in AD [38, 53] and our blots reveal significant accumulation of phosphorylated tau in the AD synaptoneurosomes and none in the LBC1936 samples (Fig. 7e). Synapses are metabolically demanding structures, requiring mitochondria in close proximity to maintain physiological function. Staining our synaptoneurosomes for the mitochondrial marker VDAC1 revealed the presence of synaptic mitochondria in all samples, with similar levels in most samples (Fig. 7f). However, strikingly higher levels were found in LBC1936 BA41/42 and AD EC compared to their matching sample (Fig. 7f).

**Fig. 8:**
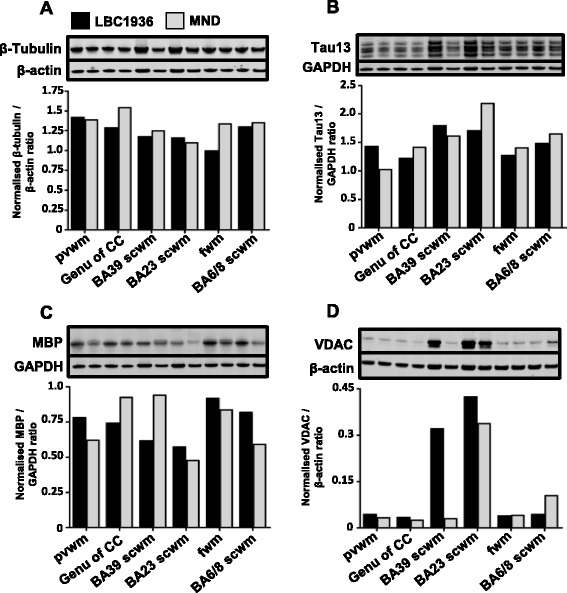
Assessment of axonal integrity and pathology using crude homogenate western blotting. **a** Representative β-III-tubulin (55 kDa) blot of five LBC1936 (black bars) and five MND (grey bars) white matter crude homogenates. **b** Total tau (Tau13; 45–60 kDa) blot of LBC1936 and MND white matter homogenates. **c** Representative MBP (23 kDa) blot of LBC1936 and MND homogenates. **d** VDAC (29–32 kDa) blot of LBC1936 and MND white matter samples. GAPDH (36 kDa) or β-actin (42 kDa) was run as a loading control. Histogram represents the mean of three experimental repeats and the bars represent the quantification of the bands directly above

The *in vivo* MRI scans from the LBC1936 participant revealed potential underlying white matter pathology (Fig. 2). Crude homogenates of white matter regions from the LBC1936 and a motor neuron disease (MND) brain were tested for axonal tract integrity by staining for structural proteins. Axonal tubulin and tau levels were similar between cases (Fig. 8a, b), however in a number of regions myelin basic protein (MBP) levels were markedly variable (Fig. 8c). Mitochondrial levels varied dramatically between white matter regions, with most expressing low levels of VDAC1 (Fig. 8d), however in the BA39 and BA23 sub-cortical white matter samples the LBC1936 expressed markedly higher levels than the MND samples (Fig. 8d).

In summary, the western blots reveal generally good synaptic and axonal integrity with low levels of Alzheimer’s pathology in the LBC1936 brain homogenates, in line with our observations from the immunohistochemical staining.

### Array tomography

**Fig. 9:**
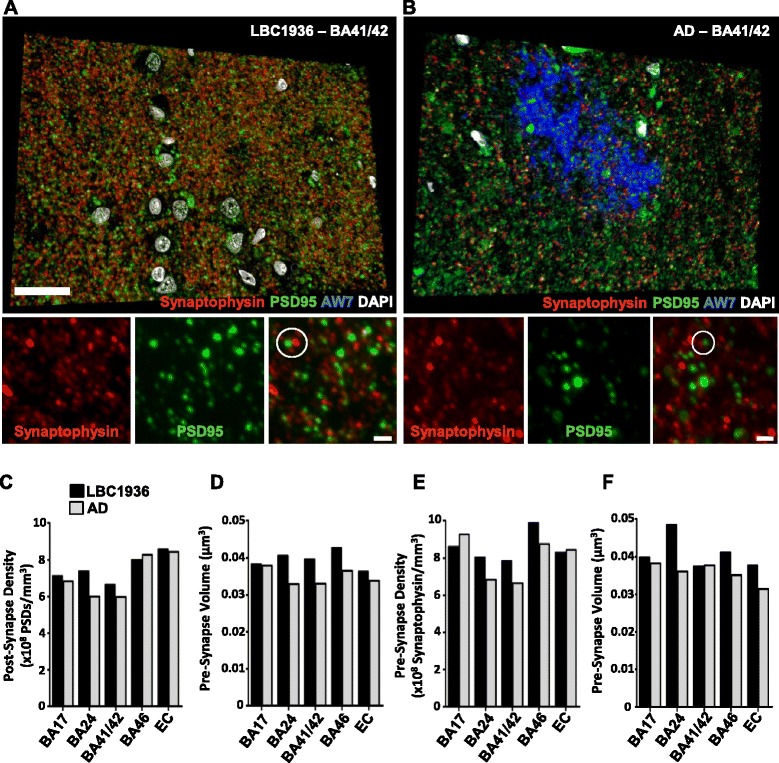
Assessing synaptic densities using the high-resolution array tomography approach. **a** Representative 3D reconstruction of thirty 70 nm sections from the LBC1936 BA41/42 cortical tissue, stained for synaptophysin, PSD95, AW7 and DAPI. Note the lack of AW7 staining, but strong synaptic labeling. Panels at the bottom show the individual synaptic channels from one of the analysed crops from BA41/42. The white circle highlights a synaptic “snowman”. **b** Representative 3D reconstruction of twenty-six 70 nm sections from the LBC1936 BA41/42 cortical tissue, stained for synaptophysin, PSD95, AW7 and DAPI. Note the weak synaptic labeling and large AW7-positive amyloid plaque. Large scale bar = 25 μm, small scale bar = 1 μm. **c** Histogram summarizing the PSD density values from 5 cortical regions from the LBC1936 brain (black bars) and an AD brain (grey bars). **d** Histogram summarizing synaptophysin densities from the LBC1936 brain and an AD brain. **e** Histogram summarizing the PSD volume data from 5 cortical regions from the LBC1936 brain and an AD brain. **f** Histogram summarizing the synaptophysin volume data from the LBC1936 cortex and an AD cortex. Histograms represent the median of all values generated from all individual crops within that region

**Fig. 10:**
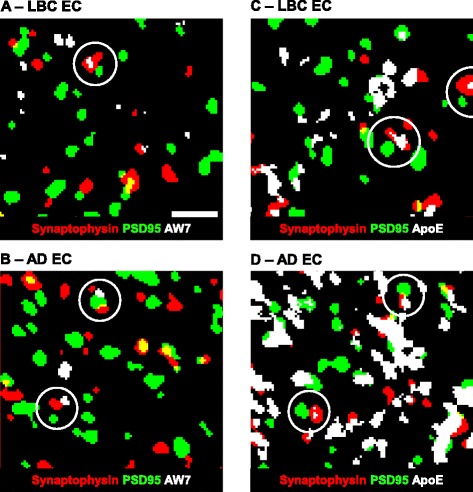
Using array tomography to assess the presence of synaptotoxic proteins. Representative images from a single region of interest (crop) captured within the LBC1936 EC (**a** + **c**) or the AD EC (**b** + **d**). Each image is a single plane from a 3D stack, which has been thresholded/binarised and single-slice objects removed to eliminate background. Sections were stained for synaptophysin, PSD95, and AW7 (**a** + **b**) or ApoE (**c** + **d**). Synaptically located staining is highlighted with white circles. **c** Scale bar = 2 μm

**Fig. 11:**
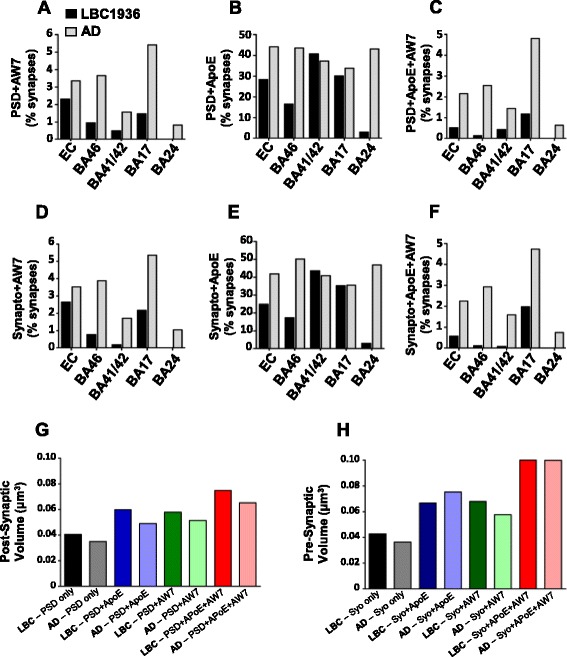
Analysing the synaptic localization of β-amyloid and ApoE. **a** Histogram showing the percentage of synapses that exhibits co-localization between PSD95 and AW7 in the LBC1936 (black bars) and AD (grey bars) samples. The percentage of co-localization between PSD95 and ApoE in the LBC1936 and AD samples is shown in (**b**) **c** The percentage of co-localization between PSD95, ApoE and AW7 in the LBC1936 and AD samples. **d** Histogram showing the percentage of synapses that exhibits co-localization between synaptophysin and AW7 in the LBC1936 and AD samples. The percentage of co-localization between synaptophysin and ApoE in the LBC1936 and AD samples is shown in (**e**). **f** The percentage of co-localization between synaptophysin, ApoE and AW7 in the LBC1936 and AD samples. **g** Histogram representing the median post-synapse (PSD) volume of all synapses from all cortical regions, positive for PSD alone, or combinations of PSD ± pathology as noted on x-axis. **h** Histogram representing the median pre-synapse (synaptophysin) volume of all synapses from all cortical regions, positive for synaptophysin alone, or combinations of synaptophysin ± pathology as noted on x-axis

Synapses from our regions of interest were labelled pre- and postsynaptically with synaptophysin and PSD95 (Fig. 9a, b) and the presence of two further proteins of interest (β-amyloid and ApoE) assessed. In total, 1500 images were analyzed, amounting to almost 162, 000 synapses. Synapses were typically observed as “snowmen” with PSD95 and synaptophysin puncta found directly opposed (see white circles in Fig. 9a, b). PSD95 densities were quite similar in most of the regions analyzed between cases, however densities in BA24 and BA41/42 appear higher in the LBC1936 case compared to the AD brain (Fig. 9c). The volume of PSD-positive compartments varied slightly between regions yet globally appeared larger in the LBC1936 brain (Fig. 9d). The variability in synaptophysin density between regions and cases, largely matched the PSD95 values, with higher densities found in BA24, BA41/42 and BA46 in the LBC1936 brain (Fig. 9e). Synaptophysin puncta volumes were larger in the LBC1936 BA24, BA46 and EC regions, but of similar size in BA17 and BA41/42 (Fig. 9f). To assess whether the synaptotoxic proteins β-amyloid and ApoE are found in synapses of the cognitively normal LBC1936 case, 3D co-localization analysis was performed (Figs. 10 and 11). A very rare population of synapses in the LBC1936 brain (<3 %) contained β-amyloid either pre- or post-synaptically (Figs. 10a and 11a,d), with a higher percentage of AD synapses (0.6 % in BA24 to 5.1 % in BA17) positive for β-amyloid (Figs. 10b and 11a, d). ApoE has been described in human synapses previously by our group [54] and that finding is confirmed here. In the LBC1936 case, the percentage of ApoE-positive synapses ranged from approximately 3 % in BA24, to 40 % in BA41/42 (Figs. 10c and 11b,e). Overall, the percentages of ApoE-positive synapses were higher and much less variable in the AD brain, ranging from approximately 33 to 50 % (Figs. 10d and 11b, e). Interestingly, there was a very rare population of synapses that contained both of these synaptotoxic proteins. Levels were very low in the LBC1936 brain ranging from almost 0 % in BA24, to ≈ 2 % in BA17 (Fig. 11c, f). Higher levels were observed in the AD brain, ranging from 0.5 % in BA24 to 4.8 % in BA17 (Fig. 11c,f). Our array tomography approach can also be used to calculate synaptic volumes, which can be used to assess changes in the pre- and post-synapse with and without pathological burden (Fig. 11g, h).

**Fig. 12:**
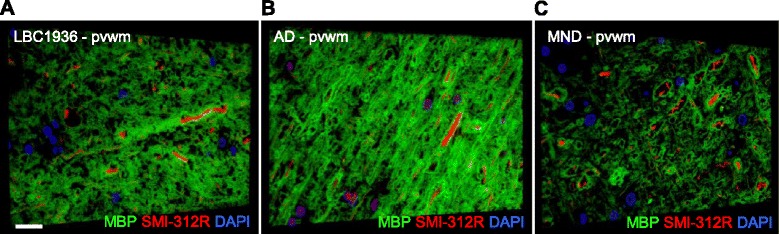
Assessing white matter integrity with the super-resolution array tomography approach. Representative 3D reconstructions of twenty-five 70 nm sections from the LBC1936 pvwm tissue (**a**), twenty-nine 70 nm sections from the AD pvwm tissue (**b**) and fourteen 70 nm sections from the MND pvwm tissue (**c**), all stained for SMI-312R, MBP and DAPI. Scale bars = 15 μm

Array tomography can also be used to build a 3D image of larger structures, such as axons. Therefore we also prepared periventricular white matter tissue (pvwm) for arrays and stained them with antibodies against axonal filaments (SMI-312R) and myelin basic protein (MBP). As can be seen in Fig. 12a and b, large caliber axonal fibers are evident in the LBC1936 and AD tissue and these are tightly wrapped in high density myelin. However, in the MND sample (Fig. 12c) the axonal fibers appear dysmorphic and loosely wrapped in low levels of myelin, when compared to the LBC1936 tissue (Fig. 12a).

In summary, our array tomography analysis provides an unprecedented wealth of detailed information on the underlying density and protein constitution of synapses in the post-mortem brain.

### Electron microscopy

**Fig. 13:**
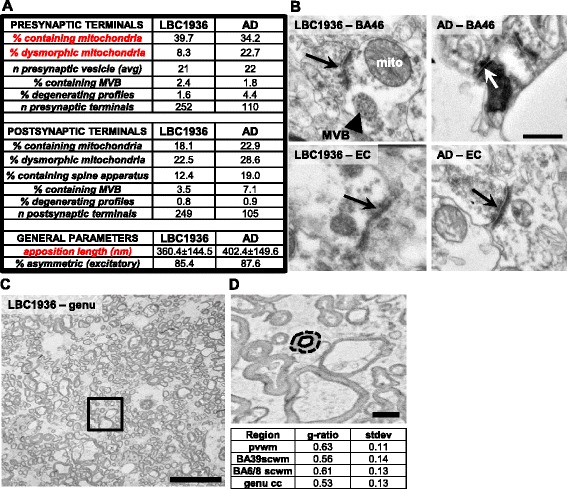
Assessment of synaptic and axonal ultrastructure using transmission electron microscopy. **a**. Table summarizing the pre- and post-synaptic parameters measured in the LBC1936 and AD brains at the EM level. Significant differences are highlighted in red. **b**. Representative electron micrographs from BA46 and EC from the LBC1936 and AD brains. Arrows point to the synapse from the pre-synapse. White arrow highlights an electron dense degenerating synapse. Mito = mitochondria, MVB = multi-vesicular body. **c**. Representative electron micrograph from the LBC1936 genu of the corpus callosum sample, showing axons cut in cross-section. **d**. Higher magnification of the boxed section in (**c**), showing how g-ratio is determined in each sample. The perimeter is draw around the internal axon cross-section (solid line) and divided by the perimeter of the axon + myelin cross-section (dashed line). G-ratio values from four white matter regions are found in the adjoining table

Images were captured from select regions in both the LBC1936 and AD brain and numerous synaptic morphological parameters were analyzed. In total, more than 350 synapses were identified as having a clear postsynaptic density and at least 3 pre-synaptic vesicles. Within individual brain regions, no differences were observed in any parameter due to the low numbers involved, however when all synapses were combined within each case, differences emerged. The LBC1936 synapses contained more presynaptic mitochondria (LBC1936 = 39.7 %, AD = 34.2 %; Chi^2^ test *p* = 0.009) and most of those present appeared of normal morphology, whereas the mitochondria found in the AD synapses were more frequently dysmorphic (LBC1936 = 8.3 %, AD = 22.7 %; Chi^2^ test *p* = 0.031; Fig. 13a). Normal mitochondria generally had a clearly defined oval shape, with distinct and intact internal cristae. Dysmorphic mitochondria had fragmented internal cristae, were often electron dense and exhibited a torturous morphology. However, other pre-synaptic parameters such as vesicle number, presence of multi-vesicular bodies and electron-dense degenerating profiles, although rarely observed, were similar between both cases (Fig. 13a, b). Furthermore, no differences were observed in any of the parameters analyzed in the post-synapse (Fig. 13a). Intriguingly, the apposition length (the length of the pre- and post-synaptic membrane directly opposing each other) was slightly longer in the AD brain (LBC1936 = 360 ± 144.5, AD = 402.4 ± 149.6; Mann–Whitney *U* test *p* = 0.006; Fig. 13a), despite smaller volumes of pre and post synapses detected by array tomography. This increase in apposition length could be a mechanism to try and compensate for shrinking synaptic volume. Electron microscopy can also be used to analyze white matter integrity (Fig. 13c) by measuring axon diameter, myelin thickness and the ratio of both known as the “G-ratio” (Fig. 13d). Measuring the G-ratio from four distinct white matter tracts of the LBC1936 brain revealed good axonal and myelin integrity, with G-ratio values falling remarkably close to the theoretically optimal value of 0.6 [55].

In summary, our electron microscopy approach reveals remarkable synaptic preservation, allowing us to investigate ultrastructural changes in human synapses, post-mortem. We detected changes in pre-synaptic mitochondria and apposition length in our AD brain and also the presence of degenerating profiles in the LBC1936 brain.

## Discussion

This study has, for the first time, described an experiment spanning almost 70 years, detailing an individual’s childhood and ageing cognitive performance and correlating this with *in vivo* imaging and post-mortem analysis to the level of individual axons and synapses. This proof-of-principle study reinforces the impact of the well-characterized LBC1936 cohort and showcases the depth of anatomical data we can gather post-mortem, to feed into this ever-expanding project, aiming to unravel the physiological processes involved in brain ageing. A significant strength of our protocol is the ability to sample and analyze many distinct cortical, subcortical and white matter regions of the brain, in relative speed, with our biochemical and array tomography approaches. For example, the Religious Orders Study and the Rush Memory and Ageing Project, generally analyze post-mortem pathology in a small number of brain regions (midfrontal gyrus, the superior temporal gyrus, the inferior parietal gyrus, and the entorhinal cortex) and extrapolate global burdens from their average [15, 56], thus missing detailed regional variation in brain change. Given the heterogeneous clinical representation of normal and pathological ageing [57, 58], it’s critical to assess the underlying brain structure from as wide an array of regions as possible to be able to fully understand the presenting phenotype.

Post-mortem pathological assessment is the only definitive way to confirm a premorbid diagnosis of Alzheimer’s disease, however this approach has proved ineffective for categorizing patients with no or mild premorbid cognitive impairment, as pathological burden varies dramatically within these cohorts [59]. This has led to a cognitive reserve theory, in which some people retain impressive cognitive function into old age, despite a heavy pathological load. The hypothesis states that people with high cognitive function throughout life (high IQ, high level of education, extensive literacy and complex social groups) can flexibly and efficiently draw processing power from other brain regions and thus retain general cognitive ability, despite underlying pathology [60]. Some of these traits have been shown to positively influence cognitive ageing in our Lothian Birth Cohort 1936 [17]. One potential strategy to help those people with pathology and cognitive decline is to remove the pathological burden allowing the brain to function more efficiently. This has been the primary goal for Alzheimer’s pharmacotherapy for many years, but unfortunately has proved spectacularly unsuccessful with a 99.6 % failure rate [61]. Therefore, new therapeutic strategies are needed and synapses or synaptic function may represent a novel approach as synapse loss is the strongest correlate with cognitive decline [62].

This enforces the importance of studying synapse density, volume and pathology in ageing cohorts such as the LBC1936, as it will undoubtedly provide valuable insight into the processes involved in synapse loss and it’s effect on brain structure and function. Our array tomography approach can reliably detect synaptic density differences between our non-demented LBC1936 brain and the AD brain (Fig. 9) and will be an important addition to the LBC1936 study as more tissue becomes available. The addition of more cases will allow us to test the correlation between cognitive performance and synaptic density in the brain regions thought to be important for specific cognitive tasks. Furthermore, synaptic density measurements can be compared with intrinsic pathology load to assess whether an inverse correlation exists or whether synapse loss is independent of detectible pathology. Without having robust data from young brains, we cannot predict how well synaptic density is preserved in the non-demented, aged LBC1936 brain, but we can conclude that compared to a pathologically aged Alzheimer’s brain there appears to be synaptic preservation.

Given the range of visible pathologies in the MRI scans from the LBC1936 cohort (Fig. 2), it will be interesting to see if these affect neuronal connectivity. Does dramatic cortical thinning mean dramatic synapse loss, or does the brain compensate by increasing connections between cells? In a seminal paper on the subject [63] DeKosky and Scheff describe cortical thinning and significant synapse loss in autopsied AD brains, suggesting that these brains don’t compensate by increasing synapse number, but interestingly the authors reported an increase in synaptic apposition length. This was also observed in our EM analysis (Fig. 13) but was not replicated in the array experiments revealing a smaller synaptic volume in the AD samples (Fig. 9). Array tomography cannot resolve ultrastructural detail, such as apposition length, but does allow high-throughput analysis of thousands of synapses and yields information about the protein composition of synapses. The presence of toxic amyloid species in synapses results in shrinkage and synaptic breakdown [64] and given the heavy amyloid burden in the AD brain (Fig. 4) it’s likely that some synaptic damage is present, which may explain our array tomography results. Indeed, we find a higher burden of synaptic amyloid in all AD samples compared to the LBC1936 brain (Fig. 11).

We have previously shown that possessing an e4 allele of the apolipoprotein E (APOE) gene correlates with increased cognitive decline in older age [24] and it confers a higher risk of developing Alzheimer’s disease [65]. APOE genotyping revealed the LBC1936 participant was APOE e3/e3 and the AD case was APOE e3/e4. We have previously shown that the ApoE protein is found in synapses in human brain [54]. Here, we found the LBC1936 cortex contained approximately 25 % of ApoE-positive synapses, compared to approximately 40 % in the AD brain (Fig. 11), strikingly similar to the 35 % of ApoE-positive synapses reported previously [54]. Finally, greater ApoE staining in the AD case associated with more amyloid at the synapse (Fig. 11). Amyloid was found at a subset of synapses in both cases, however the exact form of amyloid (monomer, polymer or fibril etc.) was not identified. The antibody we used in this study (AW7) labels all forms of amyloid. The most synaptotoxic form has yet to be conclusively revealed, although we have data suggesting multiple oligomeric forms of amyloid are present at synapses (data not shown). All of these findings match our previous results in human brain and demonstrate that our array tomography approach has the ability to reveal synaptic density and protein constitution of human synapses.

Our results show that the cognitively-healthy LBC1936 brain has more synapses than the AD brain and a lower pathological load; however some synapses in the LBC1936 brain contained amyloid, which might predispose them to degeneration. As synapses degenerate the spatial control of proteins is lost and the synapse fills with excess protein and debris, which renders the terminal electron dense under an electron microscope. We discovered a higher incidence of electron dense synapses in the AD brain than the LBC1936 samples, however some degenerating synapses were found in the cognitively-healthy LBC1936 brain. This could represent the small percentage of synapses that contain amyloid from our array tomography studies, which have reached the point of degeneration. However, without a young brain as a control it is difficult to confirm this.

The LBC1936 study has revealed that white matter integrity correlates with cognitive performance [66, 67], therefore detailed analysis of white matter at post-mortem will aid in the identification of visible abnormalities and hyperintensities observed during *in vivo* imaging (Fig. 2), which are currently unexplained [68, 69]. Our biochemistry approach allows a quick evaluation of gross protein changes in white matter tracts, which can then be resolved to sub-micron resolution using array tomography, in the hope to correlate *in vivo* observations with single cell alterations in protein composition. As shown in Fig. 8, MBP levels were quite variable between regions and cases. This could represent interesting differences in clinical and sub-clinical alterations in myelin physiology, or could be explained by post-mortem degradation of MBP [70]. Further work is required to more accurately interpret these findings and ensure we use the correct measures of axonal integrity in future studies. Array tomography can build a useful 3D representation of axons and surrounding myelin to assess the spatial distribution of key proteins in axonal integrity. For example, in future studies we can accurately measure numerous paranodal proteins found in the critical region adjacent to the Nodes of Ranvier, containing clustered ion channels and adhesion proteins, which is a vulnerable region following axonal injury or stress [71]. Electron microscopy reveals the structure of membranes in exquisite detail and can be used to calculate axonal myelin coverage. It has been predicted that the ideal G-ratio should be 0.6 [55] and we found in our LBC1936 samples that G-ratios from distinct white matter regions were remarkably close to this figure. This suggests our LBC1936 has good myelin integrity and seems to associate well with their strong cognitive performance. Greater understanding of white matter integrity in ageing brains will significantly enhance our understanding of age-related brain dysfunction and may lead to alternative therapeutics for prolonging cognition into older age.

The Edinburgh Brain Bank is currently in the process of generating a grading system for characterizing the extent of small vessel disease and different types of infarcts (micro-infarcts, large vessel infarcts and lacunar infarcts). Cognitive decline correlates strongly with the presence of underlying cerebrovascular disease [72, 73] and given the history of stroke in our LBC1936 patient in their later years and the presence of small old infarcts in the MR images, future characterization of all cases will involve assessment of the vasculature integrity and presence of localized infarcts. This will provide valuable information on the role of the cerebral vasculature on brain function and significantly strengthen the academic potential of the LBC1936 study.

## Conclusion

Ultimately, this study showcases the remarkable wealth of information we can generate post-mortem fromthe LBC1936 donors. As more tissue becomes available we will be able to draw unique correlationsbetween underlying pathology to the level of individual synapses with premorbid longitudinal cognitiveperformance. Along with the extensive biochemical, histopathological and imaging data here, geneticfactors, biomarkers and psycho-social factors can all be obtained from individuals to drive real progress inour understanding of the factors involved in normal cognitive ageing.

## Additional files

Additional file 1: Figure S1. Summary of the array tomography protocol. Numbered step-by-step flowchart summarizing the steps involved in performing array tomography. Loosely separated into three sections; tissue preparation (blue), imaging (green) and image analysis (orange). A full, detailed protocol can be found in [34]. All ImageJ and MATLAB scripts are available on request. (PPTX 19 kb) Additional file 2: Figure S2. Reference key for the semi-quantitative neuropathology scoring. Representative images showing the range of staining observed in the brains of our two cases. H + E stain is shown to highlight the clear border between the grey and white matter (white arrows), which was used to calculate cortical thickness. BA4 “score -” represents no staining. Sections were given a “score +” if any plaques were found. Scores “++” and “+++” represent clear increases in amyloid burden. AT8 “score -” represents no staining. Sections were given a “score +” if AT8-positive neurites were found, but no somatic tangles. Score “++” represents strong neuritic staining and a small number of somatic tangles. Score “+++” represents strong diffuse neuritic labeling and frequent somatic tangles. TDP-43 “score -” is given if the vast majority of cells express TDP-43 only in the nucleus. Score “+” is given if the majority of cells express TDP-43 in the nucleus, but also diffuse cytoplasmic labeling. Score “++” means the nucleus is mostly clear of TDP-43 staining and strong, diffuse cytoplasmic staining is evident. Score “+++” is given if the nucleus is completely clear and the cytoplasm contains strongly labeled aggregates. CD68 is not scored as “-” because microglia are always present. Score “+” represents numerous CD68+ cells scattered through the section. Score “++” is given if larger, complex cells with very dark staining are found. Score “+++” is given if the section contains a majority of large, complex and heavily stained cells. GFAP is not scored as “-” because astrocytes are always present. Score “+” represents diffuse but weak staining of astrocytic processes and cell soma. Score “++” is given if a few strongly labeled cell soma are evident, along with heavily labeled processes. Score “+++” is given when a larger number of strongly labeled cells are present. Ubiquitin is not scored as “-” because ubiquitin is always present. Score “+” represents the appearance of small cellular inclusions. Score “++” is given if ubiquitin-positive tangles are found in some cell soma and neurites. Score “+++” is given if strongly labeled cellular aggregates are present. Large scale bars = 150 μm and inset bars = 30 μm. (PPTX 16598 kb)
