# Erratum to: Post-mortem brain analyses of the Lothian Birth Cohort 1936: extending lifetime cognitive and brain phenotyping to the level of the synapse

**DOI:** 10.1186/s40478-015-0244-9

**Published:** 2015-12-09

**Authors:** Christopher M. Henstridge, Rosemary J. Jackson, JeeSoo M. Kim, Abigail G. Herrmann, Ann K. Wright, Sarah E. Harris, Mark E. Bastin, John M. Starr, Joanna Wardlaw, Thomas H. Gillingwater, Colin Smith, Chris-Anne McKenzie, Simon R. Cox, Ian J. Deary, Tara L. Spires-Jones

**Affiliations:** Centre for Cognitive and Neural Systems, University of Edinburgh, 1 George Square, Edinburgh, EH8 9JZ UK; Centre for Integrative Physiology, University of Edinburgh, Hugh Robson Building, George Square, Edinburgh, EH8 9XD UK; Centre for Cognitive Ageing and Cognitive Epidemiology, University of Edinburgh, 7 George Square, Edinburgh, EH8 9JZ UK; Medical Genetics Section, University of Edinburgh Centre for Genomic and Experimental Medicine and MRC Institute of Genetics and Molecular Medicine, Western General Hospital, Edinburgh, EH4 2XU UK; Centre for Clinical Brain Sciences, University of Edinburgh, Chancellor’s Building, 49 Little France Crescent, Edinburgh, EH16 4SB UK; Geriatric Medicine Unit, University of Edinburgh, Western General Hospital, Edinburgh, EH4 2XU UK; Department of Psychology, CCACE, University of Edinburgh, 7 George Square, Edinburgh, EH8 9JZ UK; Euan MacDonald Centre for Motor Neurone Disease Research, University of Edinburgh, Chancellor’s Building, 49 Little France Crescent, Edinburgh, EH16 4SB UK

## Erratum

The original version of this article [1] unfortunately contained several mistakes. The presentation of Table 2 and 3 was incorrect, in the HTML and PDF versions of this article. The corrected Tables 2 and 3 are given below.

**Table 2:** Primary antibody information

Western Blots				
ANTIBODY	COMPANY	CODE	Dilution	
PHF1	Peter Davies		1:500	
Tau13	Covance	MMS-520R-500	1:2000	
GAPDH	Abcam,	Ab8245	1:2000	
Beta-actin	Abcam	Ab8226	1:2000	
Synaptophysin	Abcam	Ab8049	1:5000	
Beta-III-tubulin	Abcam	Ab18207	1:1000	
MBP	AbD Serotec	MCA409s	1:500	
Histone	Abcam	Ab1791	1:1000	
VDAC1/Porin	Abcam	Ab34726	1:500	
GluN2B	BD Biosciences	610416	1:500	
Synapsin	Millipore	AB1543P	1:20000	
Neuropathology				
ANTIBODY	COMPANY	CODE	Dilution	Pre-treatment
Beta Amyloid (BA4)	Dako	M087201-2	1:100	98% formic acid 5 min
Alpha Synuclein	Life Technologies	32-8100	1:200	Pressure cooker/formic acid
TDP-43	2B Scientific	CAC-TIP-PTD-MO1	1:4000	Pressure cooker/citric acid
pTau (AT8)	Thermo	MN1020	1:2500	None
Ubiquitin	Dako	Z0458	1:500	Pressure cooker/citric acid
GFAP	Dako	Z0334	1:800	None
CD68	Dako	M0876	1:100	Pressure cooker/citric acid
Array Tomography				
ANTIBODY	COMPANY	CODE	Dilution	Secondary Antibody
AW7	Dominic Walsh		1:1000	Donkey α Rabbit – AF488
Synaptophysin	Abcam	Ab8049	1:50	Donkey α Mouse – AF594
ApoE	Abcam	Ab7620	1:50	Donkey α Goat – AF647
PSD95	Abcam	Ac12093	1:50	Donkey α Goat – AF488

**Table 3:** Semi-quantitative scoring of neuropathological markers

		LBC	AD			LBC	AD
Region	Stain	Score		Region	Stain	Score	
BA9	TDP43	-	++	BA41/42	TDP43	-	+
	pTAU	-	+++		pTAU	-	+++
	BA4	+	+++		BA4	+	+++
	a-Syn	-	-		a-Syn	-	-
	GFAP	+	+++		GFAP	++	+++
	CD68	+	+++		CD68	+	+
	UBIQ	+	+++		UBIQ	+	+++
BA44/45	TDP43	+	+	EC	TDP43	+	++
	pTAU	-	+++		pTAU	+	+++
	BA4	-	+++		BA4	++	+++
	a-Syn	-	-		a-Syn	-	-
	GFAP	+	++		GFAP	+	+++
	CD68	++	++		CD68	+	++
	UBIQ	+	+++		UBIQ	++	+++
BA46	TDP43	+	++	BA17	TDP43	+	++
	pTAU	-	+++		pTAU	-	+++
	BA4	-	+++		BA4	+	+++
	a-Syn	-	-		a-Syn	-	-
	GFAP	+	++		GFAP	+	+++
	CD68	+	++		CD68	+	+
	UBIQ	+	+++		UBIQ	+	+++
BA6/8	TDP43	+	++	BA24	TDP43	+	++
	pTAU	-	+++		pTAU	-	+++
	BA4	+	+++		BA4	-	+++
	a-Syn	-	-		a-Syn	-	-
	GFAP	+	+++		GFAP	+	++
	CD68	+	+++		CD68	+	++
	UBIQ	+	+++		UBIQ	+	+++

BA9 = Prefrontal cortex, BA44/45 = Broca’s area, BA46 = Dorsolateral Prefrontal cortex, BA6/8 = Premotor cortex, BA41/42 = Superior Temporal cortex, EC = Entorhinal cortex, BA17 = Primary Visual cortex, BA24 = Anterior Cingulate cortex. “-” = no pathology, “+” = mild pathology, “++” = moderate pathology, “+++” = strong pathology. Example images for each score are found in Supplementary Figure 2.

In addition, Fig. 9 was presented incorrectly, in that the label on panel 9D should read “Post-Synapse Volume”. The correct version of Fig. 9 is also provided below.

**Fig. 9:**
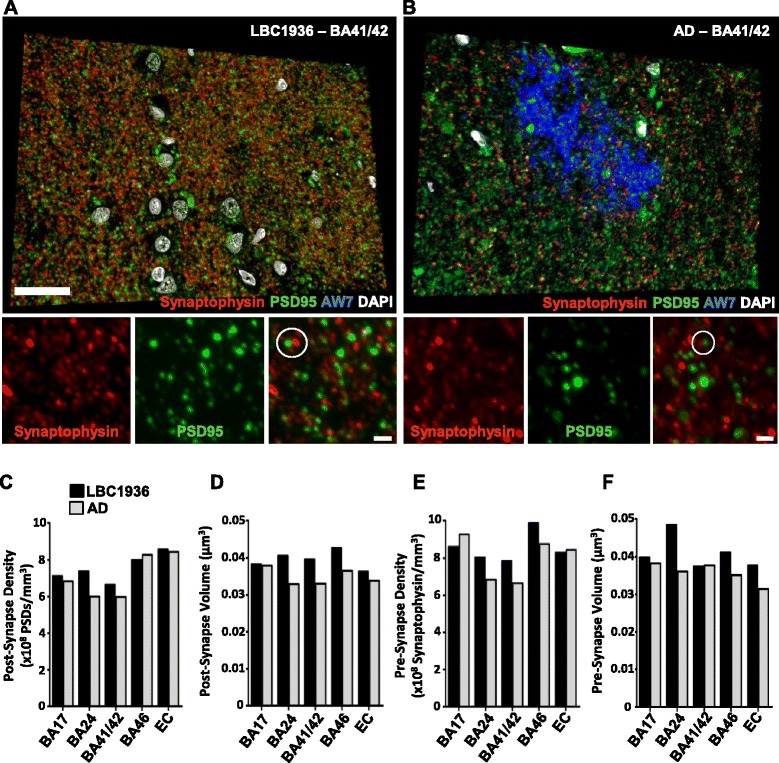
**Using array tomography to assess the presence of synaptotoxic proteins.** Representative images from a single region of interest (crop) captured within the LBC1936 EC (**A**+**C**) or the AD EC (**B**+**D**). Each image is a single plane from a 3D stack, which has been thresholded/binarised and single-slice objects removed to eliminate background. Sections were stained for synaptophysin, PSD95, and AW7 (**A**+**B**) or ApoE (**C**+**D**). Synaptically located staining is highlighted with white circles. **C**. Scale bar = 2μm.

The Conclusions and Authors Contributions sections also contain a number of typing and spacing errors that have been updated. Please see the corrected text provided below.

Lastly, the reference list has been updated to include all named authors up to the first 30.

The original article has been updated to reflect all the above changes.
