# Supplementary figures and images for: Post-mortem brain analyses of the Lothian Birth Cohort 1936: extending lifetime cognitive and brain phenotyping to the level of the synapse
Source: Acta Neuropathol Commun. 2015 Sep 4;3:53. doi: 10.1186/s40478-015-0232-0 (PMC4559320; doi:10.1186/s40478-015-0232-0)

## Slide 1
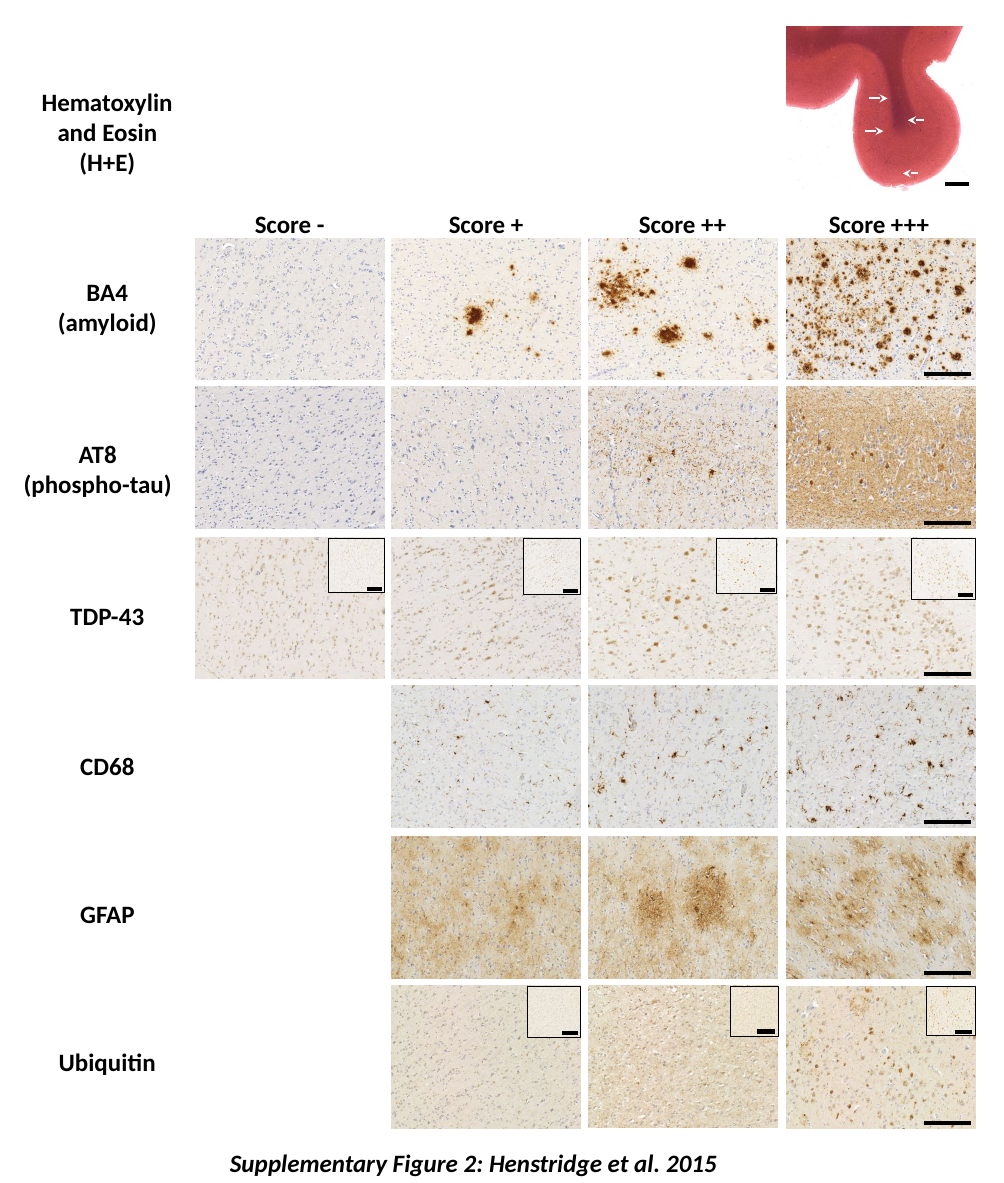

Supplement: Additional file 1: Figure S1. — Summary of the array tomography protocol. Numbered step-by-step flowchart summarizing the steps involved in performing array tomography. Loosely separated into three sections; tissue preparation (blue), imaging (green) and image analysis (orange). A full, detailed protocol can be found in [34]. All ImageJ and MATLAB scripts are available on request. (PPTX 19 kb) [file 40478_2015_232_MOESM1_ESM.pptx]

## Slide 1
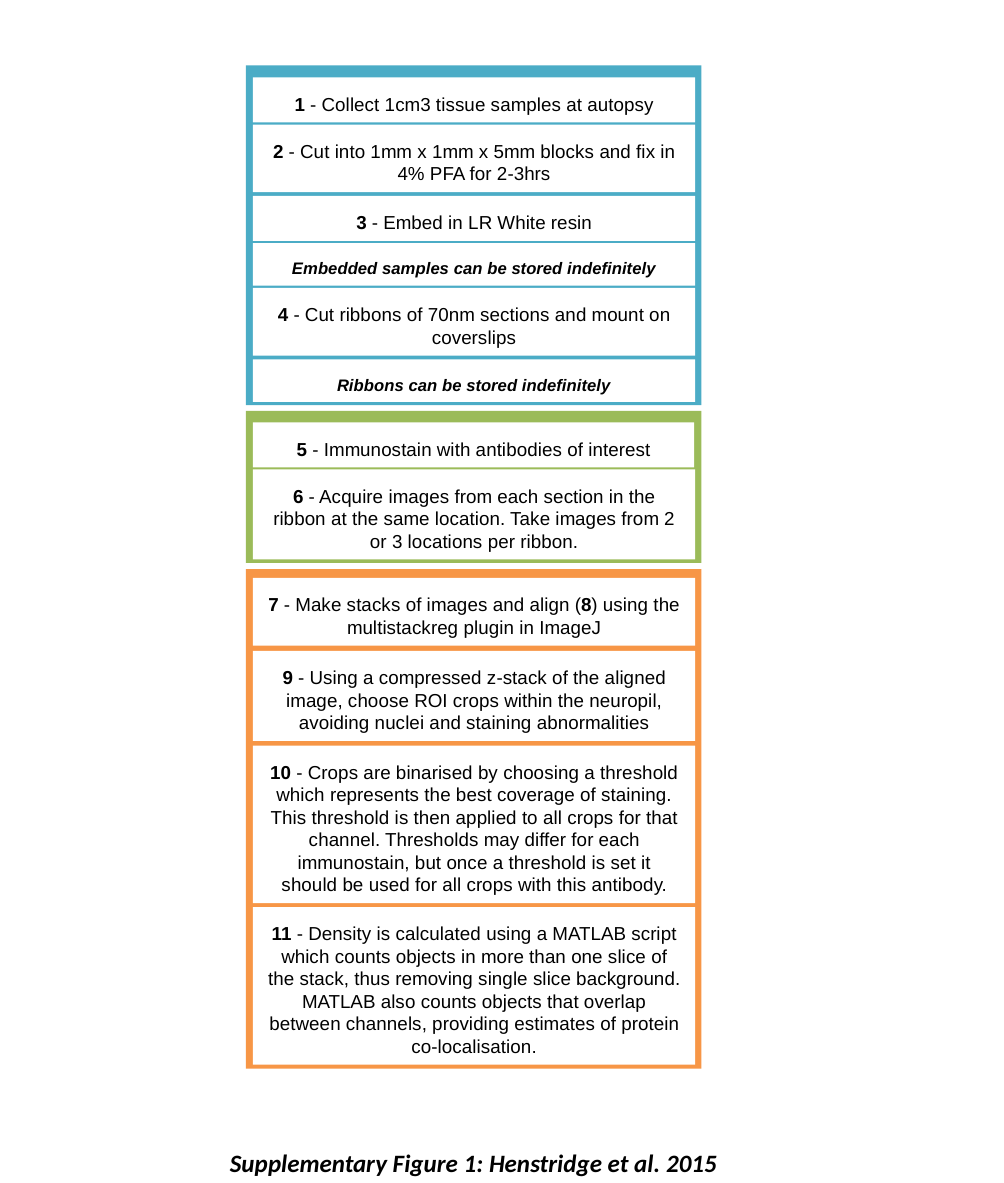

Supplement: Additional file 2: Figure S2. — Reference key for the semi-quantitative neuropathology scoring. Representative images showing the range of staining observed in the brains of our two cases. H + E stain is shown to highlight the clear border between the grey and white matter (white arrows), which was used to calculate cortical thickness. BA4 “score -” represents no staining. Sections were given a “score +” if any plaques were found. Scores “++” and “+++” represent clear increases in amyloid burden. AT8 “score -” represents no staining. Sections were given a “score +” if AT8-positive neurites were found, but no somatic tangles. Score “++” represents strong neuritic staining and a small number of somatic tangles. Score “+++” represents strong diffuse neuritic labeling and frequent somatic tangles. TDP-43 “score -” is given if the vast majority of cells express TDP-43 only in the nucleus. Score “+” is given if the majority of cells express TDP-43 in the nucleus, but also diffuse cytoplasmic labeling. Score “++” means the nucleus is mostly clear of TDP-43 staining and strong, diffuse cytoplasmic staining is evident. Score “+++” is given if the nucleus is completely clear and the cytoplasm contains strongly labeled aggregates. CD68 is not scored as “-” because microglia are always present. Score “+” represents numerous CD68+ cells scattered through the section. Score “++” is given if larger, complex cells with very dark staining are found. Score “+++” is given if the section contains a majority of large, complex and heavily stained cells. GFAP is not scored as “-” because astrocytes are always present. Score “+” represents diffuse but weak staining of astrocytic processes and cell soma. Score “++” is given if a few strongly labeled cell soma are evident, along with heavily labeled processes. Score “+++” is given when a larger number of strongly labeled cells are present. Ubiquitin is not scored as “-” because ubiquitin is always present. Score “+” represents the appearance of small cellular inclusions. S [file 40478_2015_232_MOESM2_ESM.pptx]
